# Exploring the Extent of Variance in the Development, Prognosis, and Outcome Between Primary and Secondary Cardiac Tumours: A Systematic Review

**DOI:** 10.7759/cureus.93763

**Published:** 2025-10-03

**Authors:** Aahana Nigam, Sandeep Sekar Lakshmisai, Priyanka Sakarkar, Roshitha S Bheemaneni, Evangeline C Nwachukwu, Pousette F Hamid

**Affiliations:** 1 Cardiology, Trinity College Dublin, Dublin, IRL; 2 Department of Medicine, SRM Prime Hospital, Chennai, IND; 3 General Surgery, Princess Royal University Hospital, Orpington, GBR; 4 Gastroenterology, AdventHealth Ocala, Ocala, USA; 5 Internal Medicine, Gandhi Medical College, Hyderabad, IND; 6 Health Emergency Preparedness and Response Department, Nigeria Centre for Disease Control, Abuja, NGA; 7 Neurology, Ain Shams University, Cairo, EGY; 8 Neurology, California Institute of Behavioral Neurosciences and Psychology, Fairfield, USA

**Keywords:** cardiac tumour prognosis, metastatic cardiac tumours, paediatric cardiac tumours, primary cardiac tumours, tumour microenvironment

## Abstract

This review highlights the role of genetics and cellular changes within cardiac muscle in explaining the low prevalence of cardiac tumours, and the preferential development of specific neoplastic subtypes as compared to others. The varying features of primary and secondary cardiac neoplasms are highlighted, with an extended focus on the paediatric population. By analysing past literature, medical interventions, prognostic outcomes, and pathophysiological mechanisms behind cardiac neoplasms are identified. The review adhered to the Preferred Reporting Items for Systematic Reviews and Meta-Analyses (PRISMA) guidelines and employed a thorough Medical Subject Headings (MeSH) search; 18 studies were included in the final analysis. We applied our inclusion criteria to retrieve studies in the English language published from 2000 to 2025. This review primarily includes human studies, with some evidence from animal studies, which were peer-reviewed and are available as full texts. Overall data on 628 patients with cardiac neoplasms were included to discuss the divergent properties of primary cardiac tumours (PCTs) and metastatic cardiac tumours (MCTs). The paper discusses the properties of cellular division within cardiac cells and analyses the properties of muscle cells to explain the mechanism behind the low prevalence of cardiac cancers.

## Introduction and background

Primary cardiac neoplasms have a reported incidence of 0.3-0.7% of all cardiac tumours, as identified during surgery and autopsy. In contrast, metastatic cardiac tumours (MCTs) are reported to occur up to 30 times more frequently, at a rate ranging from 2.3 to 18.3% [1,2]. Primary cardiac tumours (PCTs) are unregulated growths of tissue originating from the heart, which may be benign (PBCT) or malignant (PMCT). Benign tumours comprise up to 75% of PCTs, with myxomas accounting for 50%, and rhabdomyomas contributing to 20% of all PCTs [3]. The remaining PCTs are classified as malignant, and the most common PMCTs are angiosarcomas and unclassified sarcomas, comprising 76% of all PMCTs, followed by lymphomas and mesotheliomas [4].

On the other hand, secondary or metastatic cardiac tumours originate elsewhere within the human body, using various modalities to spread to reach the cardiac tissue. While no neoplasm has been identified to metastasise to the heart preferentially, some have been recognised to spread more frequently to the heart than others. Examples of such tumours include melanomas and primary mediastinal tumours [2]. With secondary cardiac tumours being identified in 1 in 100 autopsies, the rarity of these tumours is noted. The overall prevalence of cardiac neoplasms is significantly higher in adults; nonetheless, familial syndromes may contribute to an early presentation in the pediatric population. These tumours are associated with significant respiratory and cardiac morbidity with high mortality rates; hence, medical development is imperative in advancing cardiac oncology [5].

Current medical advancements in cardiac oncology include improvements in early cancer detection, surgical techniques, and understanding the tumour microenvironment. Histopathological analysis of cardiac tissue samples remains the gold standard for diagnosing cardiac cancer. However, the integration of cardiac MRI with other imaging modalities, such as CT and positron emission tomography (PET), has contributed to the early diagnosis of cardiac tumours [5]. Furthermore, surgical ameliorations such as the 'heart autotransplant' method involve removing the in-vivo heart, surgically removing the tumour growth ex-vivo, and reintroducing the heart into the body.

Hence, providing an alternative to chemotherapy-resistant cardiac neoplasms [6] and potentially improving prognosis can be of immense value. Nonetheless, while efforts are currently being made to conceptualise the tumour microenvironment and its role in targeted drug development [7], little is known about the pathophysiology of cardiac tumour growth and its impact on clinical outcomes. Therefore, this systematic review aims to explore any correlations between the cardiac tumour microenvironment and its impact on neoplastic growth and subsequent clinical outcomes. This includes the identification of morphological, pathophysiological, and genetically linked tumour characteristics that directly impact the overall prognostic outcome of different cardiac tumours.

## Review

Methods

Initially, the Preferred Reporting Items for Systematic Reviews and Meta-Analyses (PRISMA) guidelines were utilised to conduct the relevant research [8], which was drawn from five different databases. These databases include PubMed Central, Research Gate, Google Scholar, ScienceDirect and BioMed Central. 

The search strategy for PubMed Central included the initial search of the key concept 'cardiac tumours', which resulted in 13,836 papers. The advanced search was utilised with the appropriate Medical Subject Headings (MeSH) 'Cardiac tumours OR Cardiac malignancy OR Cardiac neoplasm ("Heart Neoplasms/complications"[Mesh] OR "Heart Neoplasms/etiology"[Mesh] OR "Heart Neoplasms/mortality"[Mesh] OR "Heart Neoplasms/pathology"[Mesh] OR "Heart Neoplasms/physiopathology"[Mesh] OR "Heart Neoplasms/secondary"[Mesh] OR "Heart Neoplasms/therapy"[Mesh] ) AND Development ("Physiology"[Mesh]) OR Evolution OR Prognosis ("Prognosis"[Mesh]) AND Primary AND Metastatic OR Secondary'. This resulted in 490 research papers, which were further filtered by year to include research from 2000 to 2025, all written in English, further narrowing the search to 396 records for screening. The screening involved the application of inclusion/exclusion criteria to exclude or retrieve reports. The inclusion/exclusion criteria are summarised in Table 1. 

**Table 1:** Inclusion and exclusion criteria for retrieving appropriate reports through the MeSH search MeSH: Medical Subject Headings

Inclusion criteria	Exclusion criteria
Published between 2000 and 2025	Published before 2000
Reports in English	Reports in other languages
Human and animal data	Case reports
Peer-reviewed papers	-
Full-text reports	-

From PubMed Central, 21 reports were deemed appropriate for retrieval. Five reports were not retrievable, and one was removed as it was a case study, resulting in 15 reports. Of the 15 reports found on PubMed, two originated from ScienceDirect and one originated from BioMed Central. An additional three reports were included from ResearchGate and one from Google Scholar by manual screening of academic titles, utilising the same keywords highlighted in the MeSH search. Therefore, 18 reports were assessed for eligibility through quality checks. All reports were independently screened by two reviewers, with discussions being conducted to resolve any discrepancies regarding the chosen articles.

Results

**Figure 1:**
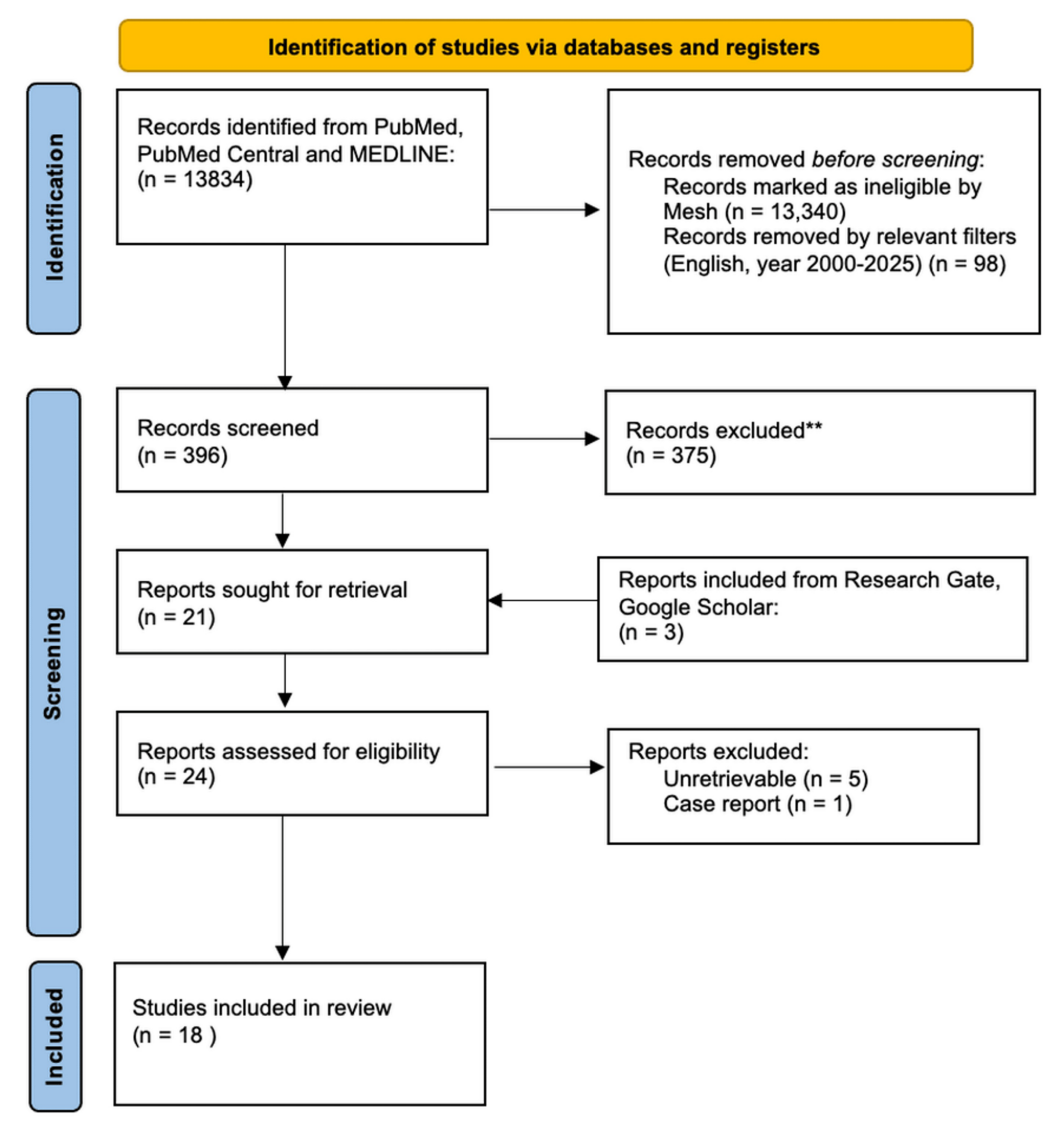
PRISMA flow chart depicting the selection of studies PRISMA: Preferred Reporting of Systematic Review and Meta-Analysis

The screening process relevant to the studies included in this systematic review is represented as a PRISMA flowchart in Figure 1 [8]. This illustrates the initial number of articles found, the number of articles removed and those added from external databases. 

The overall search elicited 18 appropriately identified reports, of which nine studies were observational, eight were traditional reviews, and one was a systematic review. The total number of patients included within this systematic review is 628. Two patients from Nomoto et al. 's study were excluded from this systematic review as they were diagnosed with a bronchogenic cyst and a thrombus. 

Quality checks were completed on each study by two other reviewers, with the relevant assessment tool used depending on the study type. The AMSTAR (A MeaSurement Tool to Assess systematic Reviews) 2 checklist was used for the systematic review [9], the SANRA (Scale for the Assessment of Narrative Review Articles) checklist evaluated traditional reviews [11,14,15,16,17,18,19,20], and observational studies were assessed through the Newcastle-Ottawa scale [10,12,13,21,22,23,24,25,26]. After the quality assessment, as shown in Tables 2-7, five studies were deemed of moderate quality, and 13 as high-quality reports. All reports were included in this systematic review. Registration on PROSPERO was not fulfilled due to time constraints.

**Table 2:** AMSTAR 2 checklist for systematic reviews The quality rating was based on the number of ‘yes’ responses achieved by the study: high quality (9-10), moderate quality (7-8), low quality (<7) AMSTAR: A MeaSurement Tool to Assess systematic Reviews

Item	Mylonas et al. [9]
Clear research question and inclusion criteria	Yes
Comprehensive literature review	Yes
Study selection and data extraction reported	Yes
Risk of bias assessed	Partial
Adequate detail on included studies	Yes
Funding sources of studies included	Partial
Appropriate methods for meta-analysis	N/A
Consideration of the risk of bias in the results	Partial
Explanation for heterogeneity	N/A
Conflict of interest disclosed	Yes
Total	8/10
Quality	Mod

**Table 3:** Newcastle-Ottawa Scale quality assessment for observational studies The quality rating was based on the number of criteria met by each study: high quality (7-9), moderate quality (4-6), low quality (1-3)

Selection	Teng et al. [10]
Representation of exposed cohort	0
Selection of non-exposed cohort	1
Ascertainment of exposure	1
Demonstration that outcome was not present at the start	1
Comparability	0
Study controls for most important factor (age)	0
Study controls for any additional factors	1
Outcome	0
Assessment of outcome	1
Was follow-up long enough for outcomes to occur?	1
Adequacy of follow-up cohorts	0
Total	6/9
Quality	Mod

**Table 4:** SANRA quality assessment for traditional reviews The quality rating was based on the number of criteria by each study, ranging from 0 to 2: high quality (10-12), moderate quality (6-9), low quality (<6) SANRA: Scale for the Assessment of Narrative Review Articles

Item	Saunders et al. [11]
Justification	2
Aims	2
Literature search	1
Referencing	2
Reasoning	2
Data presentation	2
Total	11
Quality	High

**Table 5:** Newcastle-Ottawa Scale quality assessment for observational studies The quality rating was based on the number of criteria met by each study: high quality (7-9), moderate quality (4-6), low quality (1-3)

Selection	Aboud et al. [12]	Liu et al. [13]
Representation of exposed cohort	1	1
Selection of non-exposed cohort	1	1
Ascertainment of exposure	1	1
Demonstration that outcome was not present at the start	1	1
Comparability	1	1
Study controls for most important factor (age)	1	1
Study controls for any additional factors	1	1
Outcome	1	1
Assessment of outcome	1	1
Was follow-up long enough for outcomes to occur?	1	1
Adequacy of follow-up cohorts	1	1
Total	9/9	9/9
Quality	High	High

**Table 6:** SANRA quality assessment for traditional reviews The quality rating was based on the number of criteria by each study and how strongly it was met, from 0 to 2: high quality (10-12), moderate quality (6-9), low quality (<6) SANRA: Scale for the Assessment of Narrative Review Articles

Item	Butany et al. [14]	Ekmektzoglou et al. [15]	Isaac [16]	Maleszewski et al. [17]	Campisi et al. [18]	Castillo and Silvay [19]	Taguchi [20]
Justification	2	2	2	2	2	2	2
Aims	2	2	2	2	2	2	2
Literature search	1	1	1	1	1	1	1
Referencing	2	2	2	2	2	2	2
Reasoning	2	2	2	2	2	1	2
Data presentation	2	2	2	2	2	2	2
Total	11	11	11	11	11	10	11
Quality	High	High	High	High	High	High	High

**Table 7:** Newcastle-Ottawa Scale quality assessment for observational studies The quality rating was based on the number of criteria met by each study: high quality (7-9), moderate quality (4-6), low quality (1-3)

Selection	Barreiro et al. [21]	Nomoto et al. [22]	Agaimy et al. [23]	Lin et al. [24]	Chen et al. [25]	Chen et al. [26]
Representation of exposed cohort	1	1	1	1	1	1
Selection of non-exposed cohort	1	0	0	0	1	1
Ascertainment of exposure	1	1	1	1	1	1
Demonstration that outcome was not present at the start	1	1	1	1	1	1
Comparability	1	0	0	0	1	1
Study controls for most important factor (age)	1	0	0	0	1	1
Study controls for any additional factors	1	1	1	1	1	1
Outcome	1	0	0	0	1	1
Assessment of outcome	1	1	1	1	1	1
Was follow-up long enough for outcomes to occur?	1	1	1	1	1	1
Adequacy of follow-up cohorts	1	0	0	0	1	1
Total	9/9	6/9	6/9	6/9	9/9	9/9
Quality	high	mod	mod	mod	high	high

The studies included in this review [9-26] are summarised in Table 8. The table highlights the type of study, its purpose and participant data. The overall findings related to cardiac tumour pathophysiology, clinical presentation, management and outcomes are outlined below.

**Table 8:** Summary of the included studies

Study	Type of study	Purpose of the study	Participant data	Results
Mylonas et al. 2020 [9]	Systematic Review	To explore how the tumour microenvironment (TME) differs in the heart to increase tumour resistance mechanisms	Human and animal data from secondary papers	The cardiac microenvironment is relatively more resistant to tumour formation due to the properties of stromal cells and cardiomyocyte differentiation. Stromal cell properties play a role in inhibiting tumour proliferation. Primary tumours are rare (<0.05%), while secondary tumours are more common (found in 8% of autopsies). TME characteristics such as vessel structure and stiffness are more predictive of metastasis than the size of the tumour. Mouse models revealed that mice lacking PKD1 had altered PI3K-PDK-Akt signalling, which promoted tumour metastasis and inhibited vascular development. Additionally, cardiosphere-derived extracellular vesicles in mice were shown to inhibit tumour growth. Neutrophil extracellular traps caused vascular dysfunction, impairing perfusion. Poor vascular perfusion is linked to reduced drug delivery and worse prognostic outcomes
Teng et al. 2021 [10]	Observationa (retrospective case series) study	To describe the molecular and clinical pathologic characteristics of primary cardiac synovial sarcomas and explore their outcomes	5 patients (4 males and 1 female, aged 23-48 years)	Contained all primary metastatic cardiac tumours (PMCTs), which were found to be highly aggressive with evidence of myocardial invasion. SS18 translocation was identified using fluorescence in situ hybridisation (FISH) and was used to confirm the synovial origin of the sarcomas. Ki67 was linked to increased cellular proliferation and, therefore, increased tumour aggressiveness. An overall poor prognostic outcome was achieved, with three out of five patients dying within three to twelve months post-operatively. The main factor affecting prognosis was attributed to the difficulty in achieving tumour-free margins during resection, due to anatomic limitations. Computed tomography (CT) scan and echocardiography were used to identify tumour size and location before resection. Heart transplantation was shown to have a prognostic advantage, with the patient surviving 12 months post-operatively with no evidence of disease
Saunders et al. 2024 [11]	Traditional review	To understand the tumour-resistant mechanisms underlying striated muscles, which may explain cardiac tumour resistance	Human and animal data from secondary papers	9.1% of all autopsies had evidence of metastatic cardiac tumours (MCTs), with an incidence rate of 1.23%. The lung was the most common origin of metastasis to striated muscle. Rapid blood flow to striated muscle and its contractile movement prevent tumour invasion. An intracardiac injection of tumour cells was given to mice, testing the hypothesis that muscle movement prevents metastatic seeding, resulting in the tumour cells being destroyed within five minutes. Cardiosphere cells producing extracellular vesicles have an anti-cancer protective effect and reduce the invasiveness of fibrosarcomas in mice. However, delivering capsaicin in mice to inactivate sensory nerve fibres resulted in an increased rate of cardiac metastasis
Aboud et al. 2019 [12]	Observational (retrospective cohort) study	To identify the prognostic factors contributing to survival and mortality rates following the surgical removal of primary cardiac sarcomas	17 patients (10 males and 7 females, aged 23-74 years)	The most common subtype was angiosarcoma, present in four out of 17 patients. Only one patient successfully underwent complete resection (R0), resulting in a survival time of seven years postoperatively. R0 is often difficult to achieve, due to aggressive invasion by sarcomas, but it is a strong positive prognostic factor. Immunohistology was used to identify Ki67 as a prognostic marker. Reduced Ki67 levels corresponded to better survival rates (log rank p=0.06). Regardless of metastatic disease, adjuvant chemotherapy with surgical resection consequently improved survival rates (log-rank p=0.001). Survival outcomes were not positively influenced by radiotherapy alone (p=0.42). The mean survival time was 20 months, with no drastic difference in survival rates between genders (log-rank p=0.17). The histology of tumour subtypes did not significantly impact survival
Liu et al. 2024 [13]	Observational (retrospective) study	To analyse and understand the micro-environment of cardiac myxomas	Human data compiled from secondary papers	Primary tumours were more common and often benign, while secondary tumours behaved aggressively and occurred rarely. Most secondary tumours spread haemogenously, with some directly invading the cardiac tissue. Primary tumours had cardiac predominant presentations due to mechanical interference, whereas metastatic tumours presented with vague systemic symptoms. Transthoracic echocardiography (TTE), cardiac computed tomography (cCT) and magnetic resonance imaging (MRI) were used to assess tumour morphology, location and metastasis. After resection of the relevant tumour types, primary tumours had a better prognosis and lower recurrence as compared to secondary tumours. Histology was used to confirm the tumour subtype post-resection. R0 resection was more prognostically favourable than incomplete resections. They used single-cell and spatial transcriptomics to identify the cellular origin of cardiac myxomas (CM). This revealed two main subtypes of CMs, endothelial cell-like tumour cells (ETCs) and mesenchymal stromal-like tumour cells (MTCs). Histologically, CMs had abundant myxoid stroma
Butany et al. 2005 [14]	Traditional review	To review the clinical presentation, tumour characteristics, development and outcomes of primary cardiac tumours	Human data compiled from secondary papers	Benign PCTs composed up to 75% of all tumours, with myxomas being the most prevalent. Most of these tumours arise from the atrium, predominantly the left. Secondary metastatic tumours preferentially seed in the pericardium, followed by the myocardium. Primary tumours clinically present with cardiac symptoms due to mechanical symptoms, whereas secondary cancers present with systemic symptoms. Primary malignant and secondary tumours have a poor prognosis compared to primary benign tumours. Malignant primary tumours are the most locally destructive and fatal, within months. Therefore, tumour type directly acted as a prognostic factor. TTE, CT and MRI were conducted to detect tumour location, size and metastasis. Histopathological analysis was conducted to confirm the tumour diagnosis, with aggressive histology correlating to poorer survival outcomes
Ekmektzoglou et al. 2008 [15]	Traditional review	To contrast and analyse primary and secondary cardiac neoplasms in terms of their clinical symptoms, imaging characteristics and prognosis	Human data compiled from secondary papers	75% of PCTs are benign, with cardiac myxomas being the most common. PMCTs exhibit rapid growth and tissue invasion. Secondary tumours, 20-40 times more common than PCTs, have been found to originate from the lungs, melanomas, breasts and lymphomas. Melanomas have the highest tendency to metastasise to the heart. Cardiac metastatic symptoms can be clinically silent or present late; these include effusions, tamponades and sudden cardiac death. Surgical excision is curative of primary benign cardiac lesions; however, limited in potential for malignant tumours, which have a median survival rate of less than one year. The prognosis of metastatic cardiac tumours often depends on their cancer staging, reflecting terminal late-stage disease. TTE, CT and MRI were used to determine tumour location, size and metastasis, while histological analysis was used to confirm the tumour type
Isaac, 2004 [16]	Traditional review	To investigate the clinical presentation and outcomes of human paediatric cardiac tumours	Paediatric human data compiled from secondary papers	Cardiac tumours in neonates may present post-natally as sudden cardiac death due to hydrops fetalis, or antenatally with the presence of arrhythmias. These tumours are often surgically resected; however, concomitant cardiac malformations may pose a surgical challenge. Once surgically excised, cardiac teratomas have the best survival outcomes. MCTs are extremely scarce in paediatrics; if they do occur, survival rates are minimal to zero. Tuberous sclerosis is strongly linked to the congenital presence of rhabdomyomas, which often regress over time. If persistent, controlling ventricular tachycardias during infancy is linked to reduced morbidity and improved outcomes. Hemangiomas of the heart are also documented to self-regress. TTE was conducted on all patients, with a histopathological analysis done on surgical specimens to confirm the tumour subtype
Maleszewski et al. 2018 [17]	Traditional review	To explore the clinical and radiological patterns of cardiac tumours	Human data compiled from secondary papers	Primary benign cardiac tumours are more commonly located in females. Approximately two-thirds of patients diagnosed with myxomas presented initially with symptoms associated with cardiac obstruction. One-third of myxoma patients have embolic symptoms, with an increased likelihood if the tumour is present in the left ventricle. The carney complex is responsible for 5% of myxomas. Fibromas can present as part of the Gorlin-Goltz syndrome. Additionally, immunosuppression increases the likelihood of developing cardiac neoplasms, such as metastatic cardiac lymphomas. Paediatric neoplasms are often part of a syndrome, such as tuberous sclerosis. Rhabdomyomas are the most common, followed by papillary fibroelastomas. Angiosarcomas, a primary malignant cardiac tumour, often present as a right atrial mass and an accompanying pericardial effusion. The prognosis for benign tumours is exceptional; however, it gets significantly reduced for neoplasms exhibiting malignant tendencies. Surgical debulking has been attributed to a better prognosis. While the location of a tumour is correlated to the presenting symptoms, it was not shown to affect the overall prognosis. With end-stage malignant or metastatic cardiac tumours, palliative care is recommended
Campisi et al. 2022 [18]	Traditional review	To review the clinical manifestations, management and outcomes of cardiac oncology	Human data compiled from secondary papers	Secondary cardiac tumours are 20x more common than primary, which have a prevalence of 0.18% postpartum. They found 10-24 months to be the average survival time for malignant primary tumours, despite surgical intervention and chemotherapy. Angiosarcomas were found to exhibit only six months of survival time after interventions, with high recurrence rates. Palliative care was recommended for metastatic cardiac disease. Prognosis is impacted by tumour size, morphology, intervention type and aggressiveness. Echocardiography was used to assess tumour properties, with the definitive diagnosis conducted through histopathology
Castillo and Silvay, 2010 [19]	Traditional review	To explore different cardiac neoplasms and understand their management	Human data compiled from secondary papers	Classic features to characterise a tumour's behaviour include its location, size and degree of invasiveness. Myxomas located within the left atrium can result in valvular heart disease, while those located in the right atrium can present as right heart failure symptoms. Furthermore, they can present as systemic features, with inflammatory markers posing a diagnostic delay. Fibromas commonly occur in those less than one year of age and are the most frequently removed tumours from this population. Half of these masses are associated with tuberous sclerosis. Angiosarcomas are highly malignant lesions, often diagnosed after metastasis has occurred. Lymphomas are preferentially present in those who are immunodeficient as a primary malignant tumour. Cardiac metastasis from secondary tumours occurs in 10% of cancers.
Taguchi, 2018 [20]	Traditional review	To evaluate adult malignant cardiac tumours, including their occurrence and treatment	Human data compiled from secondary papers	Primary malignant tumours were identified as sarcomas, lymphomas and malignant mesothelioma. Whereas metastatic tumours were commonly characterised as carcinomas from various origins. Primary tumours located within the pericardium are usually malignant. The haematological route is the preferred route to cardiac metastasis. The survival of patients with cardiac cancer has improved by an average of five years. Resection of tumours with positive margins had a worse prognosis than those with negative margins, averaging at 16 months post-operatively as compared to 27 months. The study used histopathology, CT images and echocardiography in their discussion
Barreiro et al. 2013 [21]	Observational study	To review surgical cardiac oncology cases in terms of their methodology and outcomes	73 patients (25 males and 48 females, aged 15-92 years)	The most common benign cardiac tumour was found to be cardiac myxoma. PBCTs that underwent surgical resection had an excellent prognosis, with a 0% one-year mortality rate and recurrence after two years. Primary malignant tumours were found to be more prevalent in males and occurred at a younger age, with undifferentiated angiosarcomas being the most prevalent in the study. The average survival for all patients with PMCT was four months, with a 100% mortality rate observed within one year. Exceptions to the high mortality rate occurred in those who received a multimodal treatment approach and/or heart transplantation. Nonetheless, despite surgery or chemotherapy, malignant tumours had poor outcomes. All patients underwent TTE imaging, with 42 patients additionally requiring a transesophageal echocardiogram (TEE). CT and cardiac MRIs were also used. Histopathology was used as the diagnostic gold standard. MRIs were found to be increasingly useful in pre-operative surgical planning and tissue identification
Nomoto et al. 2017 [22]	Observational study	To analyse the echocardiographic diagnosis, management and outcomes of primary and metastatic cardiac tumours	93 patients (55 males and 38 women, aged 51-79 years)	Primary cardiac tumours were present in 64% of the population, whereas MCTs were responsible for 36% of all cases. The most common PCTs were myxomas, followed by papillary fibroelastoma. Metastasis to the heart was found to originate from the lung, kidneys and thyroid. The most common tumour location for MCTs is the atria, followed by the inferior vena cava. The tricupid annulus was found to be a specific metastatic location for lymphomas. Benign tumours had a 100% survival rate. However, malignant tumours had an average survival rate of 113.5 months ±34.1 months, and a mortality rate of 70%.. The extent of tumour resection was important for determining overall tumour prognosis. Key echocardiographic differences to isolate malignant tumours from benign were the myocardial invasion, pericardial effusion and tumour extension to multiple chambers. Diagnosis was achieved by TTE, TEE and echocardiography. Echocardiography was found to be accurate in 80% of cases, with histopathology used as the gold standard
Agaimy et al. 2012 [23]	Observational study	To compare and contrast the clinical manifestations, treatment and prognosis of primary and metastatic cardiac tumours	9 patients (aged 28-81 years)	Of the nine patients, five had cardiac sarcomas, located on the left side of the heart in four out of five patients. The average age for women, 56.7 years, was higher than men, who had a mean age of 31.5 years. Additionally, four patients had metastatic sarcomas (MCT), with an average latency to cardiac metastasis of 109.5 months. Metastatic cardiac tumours occurred at an average age of 50.7 years, which was found to be higher than the average age of primary sarcoma development at 45.6 years. These masses were found to be difficult to excise surgically due to deep myocardial infiltration. A possible prognostic correlation was explored between tumour grade and cardiac sarcomas. All patients with primary sarcomas underwent median sternotomy, and one patient had adjuvant chemotherapy. One individual had an unresectable angiosarcoma and underwent heart transplantation. The average survival after surgical excision was 24.5 months, whereas the heart transplant patient was still alive at the time of publication. MCT patients underwent surgical resection if possible, and one patient underwent heart transplantation. Three out of four patients died within 30 months, while the heart transplant patient was still alive at the time of publication. TTE, TEE, CT and cardiac MRI were used as imaging modalities to evaluate the cardiac lesions. Once resected, histopathological analysis was done on the tumours
Lin et al. 2023 [24]	Observational study	To explore the clinical presentation and outcomes of metastatic cardiac neoplasms	41 patients (25 males and 16 females, aged 49.5-66.5 years)	Of the 41 patients diagnosed with cardiac metastasis, 12 had developed cardiac metastases at the time of their initial cancer diagnosis. The most common primary tumour with cardiac metastasis was identified as lymphoma. Most MCTs existed as singular lesions, while three of the 12 patients had three cardiac lesions. Previous cardiac disease was not found to be a contributing factor to the development of cardiac metastasis. In early disease, cardiac function was found to compensate for any damage caused by the metastasis. Echocardiography, CT and histopathological analysis were used for diagnosis. The mean survival time for patients receiving treatment was 27.3 months, whereas those without prolonged treatment survived an average of 6.6 months. When MCTs responded to treatment, the primary neoplasms were also found to be responsive
Chen et al. 2019 [25]	Observational study	To examine the clinical features, outcomes and treatment options for cardiac sarcomas	61 patients (28 males and 33 females, aged 18-79 years)	Among all sarcomas, histopathologically, angiosarcomas were identified more frequently. 39 patients had localised lesions, whereas 22 patients had developed metastasis. The average survival duration in the study was 17.5 months. Three main prognostic factors were correlated to improved survival, such as an age <65, no metastasis at initial tumour diagnosis and surgical treatment for primary lesions. Nonetheless, a high recurrence rate amongst individuals was observed despite the surgical removal of the sarcomas. Tumours identified as unresectable had the worst prognostic outcomes with a mean survival time of 8.9 months. Additionally, multimodal treatment was found to increase survival time as compared to single-modality treatment regimens. The survival times were 36.5 months as compared to 14.1 months, respectively. Tumour sizes were measured using CT or MRI, aided by histological analysis to confirm the diagnosis
Chen et al. 2023 [26]	Observational study	To analyse the prognostic influences and outcomes of primary malignant cardiac disease	329 patients (177 males and 152 females, aged 0-76 years)	The study population originated from the SEER database, which included tumour size, AJCC staging, metastasis status and histological confirmation of the lesions. PMCTs were found to affect patients ≤76 years more than those >76. Prognostic factors were determined by the size of the tumour, the development of metastasis and cancer staging. Tumours <99mm, no metastasis, use of chemotherapy and AJCC staging ≤III was found to be prognostically favourable. However, despite the use of chemotherapy, if the patients were >76 years or had distant metastasis, a poor outcome was noted. Tumour size was not found to impact the survival of those receiving chemotherapy. Finally, surgical removal of PMCTs and the use of radiotherapy were not found to improve the prognostic outcome

**Figure 2:**
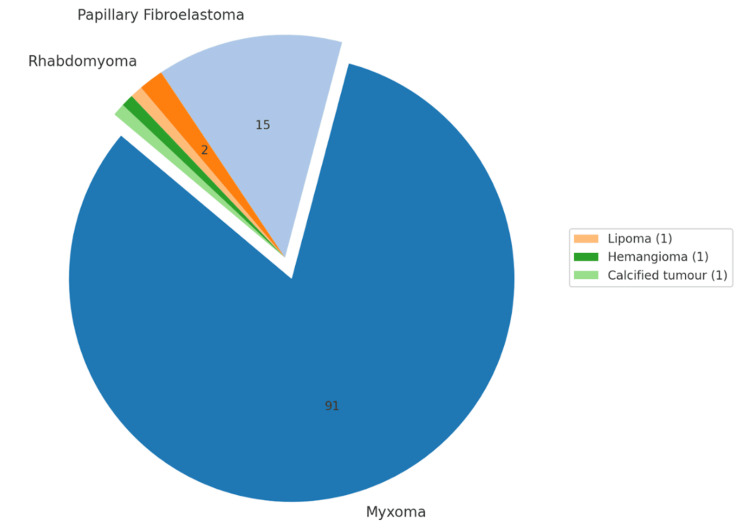
Distribution of primary benign cardiac tumours* ^*^[21,22,23] The figure was CREATED by the original authors of this article

**Figure 3:**
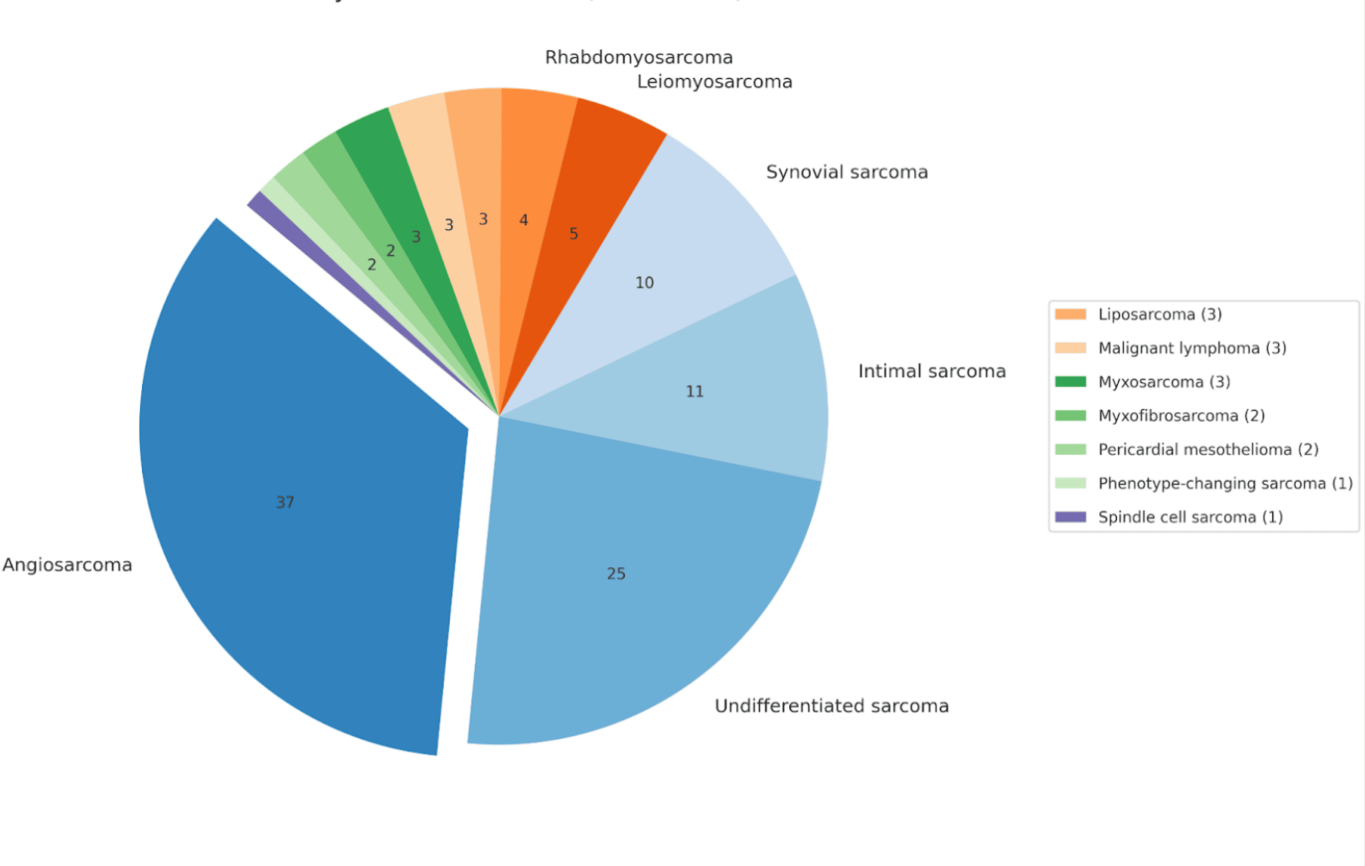
Distribution of primary malignant cardiac tumours* ^*^[10,12,21,22,23,25] The figure was created by the original authors of this article

**Figure 4:**
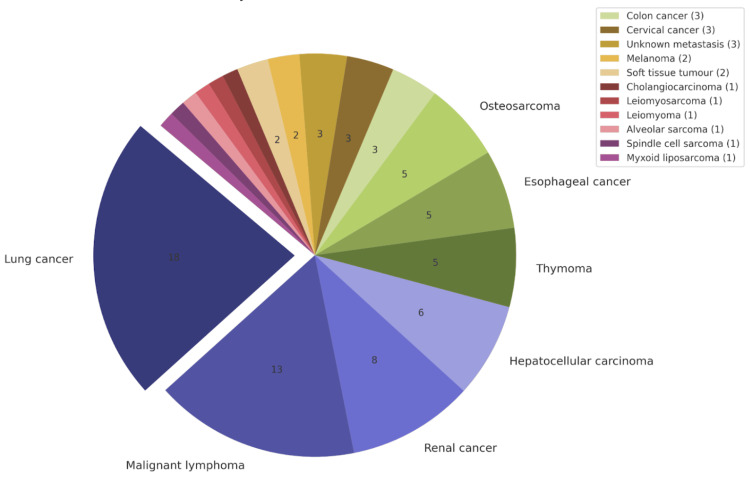
Distribution of metastatic cardiac tumours* ^*^[12,21,22,23,24]

From the studies included in this review, patient data related to cardiac neoplasms have been outlined in Tables 9-11 and Figures 2-4. The tables highlight the type of neoplasm, its prevalence in the study population, its gender and location preference. Table 9 indicates primary benign cardiac tumours included within this study, originating from sources 21-23. Table 10 contains information related to primary malignant cardiac tumours [10,12,21,22,23,25]. Table 11 illustrates metastatic cardiac tumour data [12,21,22,23,24].

**Table 9:** Combined patient information of those diagnosed with primary benign cardiac tumours* ^*^[21-23]

Primary benign cardiac tumours (PBCT)	Total patients	Gender preference	Location
Myxoma	91	Females	Left atrium
Papillary fibroelastoma	15	N/A	Left atrium, aortic valve
Rhabdomyoma	2	N/A	Left and right ventricles
Lipoma	1	N/A	N/A
Hemangioma	1	N/A	N/A
Calcified tumour	1	N/A	N/A

**Table 10:** Combined patient information of those diagnosed with primary malignant cardiac tumours* ^*^[10,12,21,22,23,25]

Primary malignant cardiac tumours (PMCT)	Total patients	Gender preference	Location
Angiosarcoma	37	Males	Left atrium
Undifferentiated sarcoma	25	Equal prevalence	Equal left and right prevalence
Intimal sarcoma	11	Female	Left-sided
Synovial sarcoma	10	Males	Right-sided, mitral valve
Leiomyosarcoma	5	N/A	Left atrium
Rhabdomyosarcoma	4	Female	Left atrium, right ventricle
Liposarcoma	3	Female	Left-sided
Malignant lymphoma	3	N/A	Right atrium, tricuspid annulus
Myxosarcoma	3	Females	Left atrium
Myxofibrosarcoma	2	N/A	N/A
Pericardial mesothelioma	2	N/A	Pericardium
Phenotype-changing sarcoma	1	Male	Left-sided
Spindle cell sarcoma	1	Females	Left atrium

**Table 11:** Combined patient information of those diagnosed with metastatic cardiac tumours* ^*^[12,21,22,23,24] IVC: inferior vena cava

Metastatic cardiac tumours (MCT)	Total patients	Gender preference	Location
Lung cancer	18	N/A	Right ventricle
Malignant lymphoma	13	N/A	Right-sided
Renal cancer	8	N/A	Right atrium, IVC
Hepatocellular carcinoma	6	N/A	N/A
Thymoma	5	N/A	N/A
Esophageal cancer	5	N/A	Right ventricle, pericardium
Osteosarcoma	5	Female	Left atrium
Colon cancer	3	N/A	N/A
Cervical cancer	3	Females	Left ventricle
Unknown metastasis	3	N/A	N/A
Melanoma	2	N/A	Pericardium
Soft tissue tumour	2	N/A	Right ventricle, pericardium
Cholangiocarcinoma	1	N/A	N/A
Leiomyosarcoma	1	N/A	N/A
Leiomyoma	1	N/A	N/A
Alveolar sarcoma	1	N/A	Left ventricle
Spindle cell sarcoma	1	Females	Left atrium, mitral valve
Myxoid liposarcoma	1	Male	Right atrium, IVC

Our systematic review included 112 primary benign cardiac tumours; myxomas were the most prevalent, followed by papillary fibroelastomas and rhabdomyomas. A total of 107 primary malignant tumours were found, with angiosarcomas being the most common, followed by undifferentiated and intimal sarcomas. Finally, 79 metastatic tumours were included in this study; the most common primary tumour to metastasise to the heart was from the lungs, followed by malignant lymphoma and renal cancers. While the total number of patients included in this study is 628, the results tables are reflective of 297 patients. The results tables did not include the data from the 329 patients in Chen et al.’s study. They located their patient population directly from the SEER database and failed to provide details on individual cases. Additionally, Lin et al. lacked the tumour specification for two patients, resulting in 39 of 41 patients from their study being included in the combined results table.

Discussion

Pathophysiology of Tumour Development

The basic tumorigenesis model uses a sequence of analogous events, such as cellular immortality through cell signalling and the evasion of apoptosis. The tumour microenvironment (TME) is produced through alterations in cellular metabolism of cancerous cells, resulting in pathophysiologic states of oxidative stress and acidosis [9]. In cardiac synovial sarcomas, hyalinisation and myxoid changes have been observed in concentrated and sparse cellular collections [10]. However, the cardiac microenvironment is known to be relatively resistant to tumour growth compared to other organ systems, reflected by the low prevalence of PCTs and MCTs within the population. A possible cancer-resistant mechanism is attributed to the stability of cardiomyocytes, which terminally differentiate and ensure low turnover rates [9].

When comparing cardiac stromal cells to those from bone marrow, adipose tissue and liver cells, cardiac cells exhibited increasing resistance against the ‘seeding’ of tumour cells and the introduction of primary tumorigenesis [9]. Recent studies involving mice have discovered poor vascular responses in the heart and kidneys in response to pathologic cell-signalling molecules such as vascular endothelial growth factor (VEGF). The inadequate response has been attributed to neutrophil extracellular traps (NETs), resulting in the occlusion of vasculature and possibly explaining the low rates of metastatic disease to the heart [9]. 

Many studies have been unable to molecularly examine cardiac myocytes. However, similar resistance to tumorigenesis has been demonstrated within skeletal muscle cells. Saunders et al. explore the mechanisms involved within striated skeletal muscle that protect the organ system from malignancy and may extend to the cardiac myocytes. One possible explanation is attributed to the high blood flow shunted into skeletal muscle cells, accompanied by the stereotyped movement of muscle, which prevents and destroys cancer cells. Hence, the cardiac environment of mice was introduced with exogenous cells to test this hypothesis, resulting in the cells being destroyed within five minutes of the intracardiac injection.

Additionally, studies on mice highlighted the role of extracellular vesicles within cardiosphere cells, which contain anti-cancer properties to reduce the invasiveness of fibrosarcomas in vitro and on xenografted human models. Therefore, heart stromal cells reduce the viability of tumour cells, either through secretory elements or direct contact. However, it has been suggested that damage to muscular environments may increase the likelihood of a TME via altered physiology, such as pH and temperature. For example, a toxic dose of capsaicin was delivered in cancer-bearing mice to inactivate sensory nerve fibres, which overall led to an increased rate of metastatic disease to the cardiorespiratory system [11]. 

While the cardiac cellular environment is relatively more resistant to tumorigenesis, recent research has identified varying factors which may contribute to cancer development. The altered histogenesis of myocytes has been associated with the cellular stromal arrangement. This may contribute to the increased vulnerability of particular anatomical sites to cancer development. A deficiency in PKD1, an initiation factor involved in the P13K-PKD-Akt pathway in mice, has been correlated to the development of chaotic vascular systems. These disorganised networks aid in translocating cancerous cells and increase the metastatic rates of systemic cancers [9].

Aboud et al. explore the prognostic factors of sarcomas and highlight the involvement of cellular markers involved in protein regulation during the cell cycle and in cell kinetics in the pathology of tumour development. Therefore, discovering the correlation between high expressions of the pKi67 marker and the increasing proliferation of cell lineages and cardiac tumour aggressiveness [12]. However, in Teng et al.’s focused study on aggressive cardiac synovial sarcomas, the pKi67 proliferation index was relatively low, with an average of 23%. Instead, prominent CD34 signalling was prevalent and limited to the vasculature, with 90% of all cases attributed to the chromosomal translocation t(X;18)(p11;q11) involving the SS18 gene on 18q [10]. Therefore, the correlation between all cardiac tumours and tumour aggressiveness, based on the expression of pKi67, may require further research to determine the specificity of the potential marker. 

While many studies have not explored the TME for varying cardiac tumours, Liu et al. highlighted the key characteristics of the PBCT cardiac myxomas (CM), which are shared with well-known malignant tumours. Two pivotal cell types were identified: endothelial tumour cells (ETCs) and mesenchymal tumour cells (MCTs). In the setting of CM, it was found that ETCs are formed by differentiated MTCs utilising key genes such as MIA, TCF4 and CCDC80. The extent of ETC differentiation was noted to impact cancer growth and the progression of disease, possibly existing as a future marker for disease burden. The promotion of cardiac stem cells into a TME was traced back to somatic mutations. Considerable genomic rearrangements within cells and the extent of cellular heterogeneity were shared features between CM and malignant tumours. Haemorrhaging was discovered to considerably affect the extent of heterogeneity observed within these cells, posing as a possible negative prognostic factor [13].

Familial Syndromes

A common source of primary cardiac tumour development has been attributed to a myriad of familial syndromes and genetic predispositions. Ekmektzoglou et al. underscore familial myxomas, which are inherited in an autosomal dominant (AD) fashion [15]. These myxomas are often a part of the LAMB (lentigines, atrial myxoma, blue naevi) and NAME (naevi, atrial myxoma, myxoid neurofibromata, ephelides) syndromes, composing the Carney complex. The Carney complex has been associated with an inactivating germline mutation in the PRKAR1A gene, which is responsible for protein phosphorylation to control gene expression [14,15,16,17]. Therefore, this results in decreased tumour-suppressing activity within cells and a contribution to tumour development. Additionally, rhabdomyosarcomas are commonly seen within tuberous sclerosis, an AD medical syndrome. It is associated with the germline loss-of-function (LOF) mutation in the tumour-suppressing genes tuberin or hamartin, disrupting the rapamycin pathway. Finally, fibromas have been traced to the AD Gorlin-Goltz syndrome caused by the LOF mutation in the PTCH1 gene. This gene is known to have tumour-suppressing functions and is transcribed as a transmembrane protein, resulting in altered cell signalling [15,16,17,18]. Collectively, these familial syndromes illustrate the degree of impact tumour-suppressing genes play in the role of cardiac tumour development.

Pathophysiology of Cancer Presentation

Direct and indirect mechanisms contribute to tumour development in cardiology. Direct mechanisms for PCT include primary cardiac or intraluminal mass growth, whereas MCTs occur through direct or hematolymphoid metastasis. Indirect mechanisms refer to those which increase the susceptibility of the myocardium to neoplastic changes. Examples include exposure to thoracic radiation, chemotherapeutic agents and the development of carcinoid or amyloid disease. Radiation causes neoplastic changes by generating reactive oxygen species (ROS), resulting in reactive chronic changes within the myocardium, such as fibrosis and hyperplasia. Finally, physiologic factors are further implicated in tumour development, for example, 20% of cardiac lymphomas occur in immunocompromised individuals [17]. 

Cardiac tumours, benign or malignant, may give rise to distressing symptoms by disturbing normal physiology. The mass may impede the intracardiac flow of blood or hinder valve function, whereas regional extension of the overgrowing tissue can result in arrhythmias or cardiac tamponades via pericardial effusions. The location of the tumour is strongly correlated with the presentation of systemic symptoms. Tumour growth in the left ventricle can embolise and obstruct distal vasculature, resulting in symptoms such as a stroke or limb ischemia [15,17]. Whereas, masses within the left atrium often mimic symptoms of mitral valve failure, and right-sided tumours reproduce signs of right heart failure [19]. 

Human Paediatric Population 

PCTs within the paediatric population commonly occur as a consequence of familial syndromes, most of which arise due to the association with tuberous sclerosis and include rhabdomyomas and lipomas [14,17]. Rhabdomyomas are the most prevalent cardiac masses in neonates and often involuted by six years of age. The behaviour of specialised spider cells within the mass expresses high intracellular activity of the ubiquitin pathway, contributing to the tumour suppressive properties and permitting self-resolution [16]. Second to rhabdomyomas, papillary fibroelastomas are frequently identified, with 80% possessing rKRAS mutations [14,17]. 

Primary and Metastatic Cardiac Tumours

Primary cardiac tumours:* *Primary cardiac tumours are classified as either mural or intracavitary, with the majority of intracavitary lesions being malignant [20]. However, the majority of PCTs are identified as benign and can be located in individuals of any age, with the average age of diagnosis at 59-65 years [21,22]. A higher prevalence is found in females, equating to 65.7% of cases according to Barreiro et al. [14,15,17,21]. The general consensus of literature states that PBCTs are predominantly located on the left side of the heart, preferentially involving the atrium, with the most common PBCT being cardiac myxomas [12,14,17,18,19,22]. Notwithstanding, Butany et al. interestingly challenge the current label of cardiac myxomas as the most common PBCT, further suggesting that papillary fibroelastomas may be better suited to the title. These small and multiple lesions are often overlooked due to practical regulations failing to require sectioning of surgically retrieved valves, and they possess the potential to embolise. Consequently, it affects patients adversely by events such as sudden death, via obstruction of the coronary vasculature [14]. 

Malignant changes in PCTs have been identified as numerous calcified centres and tissue invasion [19]. Discrepancies between the literature are seen when deciding on the most common malignant PCT. Butany et al. believe angiosarcomas are the most common, whereas Maleszewski et al. found it to be the undifferentiated high-grade pleomorphic sarcoma (UGHPS) [14,17,18,21]. Nonetheless, sarcomas have been identified as the most common PMCT [18,22]. Similarly to PBCT, UGHPS occur more frequently in females and are commonly located in the left-sided chambers of the heart [17,22]. However, it's important to note that PMCTs holistically affect males more than females [18,21] and at a younger age [21,23].

The presentation of sarcomas can range from an asymptomatic presentation to symptoms of respiratory difficulty, such as dyspnoea and exercise intolerance [23]. Pericardial effusion is a common recurring consequence of PMCTs and has been used as a diagnostic criterion if accompanied by an irregular cardiac mass [22]. Other malignant PCT may present with symptoms of heart failure, observed in 70% of patients in the study by Barriero et al., followed by dizziness, angina and new onset murmurs [21]. If PMCTs metastasise further to involve other organ systems, the hematogenous route is prioritised most regularly. Secondary metastasis has been identified mainly within the lung, followed by soft tissues such as the mediastinum [20]. 

Human Paediatric Population:* *In Isaac et al.'s literature review on neonatal and fetal cardiac tumours, primary cardiac tumours were found to be substantially more prevalent than MCTs [16]. Antenatal identification of arrhythmias was considered an early clinical sign of possible cardiac mass growth [16,17]. Rhabdomyomas are the most prevalent PCTs in neonates and comprise 60% of all PCTs, where 50% are associated with tuberous sclerosis [14, 16, 17, 18, 19]. Meanwhile, in adults, the most common PCT is identified to be myxomas [14,16,17,18]. The second most common PCT in paediatrics is the papillary fibroelastoma, which commonly occurs on the mitral and aortic valves [17]. Furthermore, fibromas are frequently identified in the paediatric population, with a third diagnosed in children younger than one year of age. They are the most commonly resected cardiac masses in kids [19]. In older children, fibromas often present similarly to rhabdomyomas on echocardiography and can be differentiated through the presence of calcifications [18]. 

Metastatic Cardiac Tumours:* *While tumours with the highest metastatic potential to the heart have been identified as melanomas [14], the most frequently identified MCTs originate from primary lung cancers, followed by lymphomas [14,15]. The current incidence of metastatic cardiac disease is 10% and continues to rise [19]. MCTs expand by either direct tissue infiltration or by haematogenous outspread, further utilising the lymphatic system to approach pericardial and myocardial tissue [14,15,17]. Metastatic disease from the abdominal cavity, including hepatocellular and renal carcinoma, has been achieved through spread via the inferior vena cava [18]. The average time to develop metastasis to the heart was 4.3 months in Lin et al.'s study. Additionally, at the time of primary tumour diagnosis, one-fourth of the patients had pre-existing cardiac metastasis [24].

In Agaimy et al.'s study, patients with metastasis from sarcomas had an average age of 50.7 years [23]. However, this does not necessarily correlate with the average age of overall cardiac metastasis; instead roughly reflects the age of risk. The most common location of MCTs is the epicardium; they can occur solitarily or as multiple lesions [15, 24]. If an MCT has extended into the myocardium, the right side of the heart has the highest propensity to host the tumour; however, the exact cardiac chamber is often debated [15,22]. The increased vulnerability to metastatic seeding on the right side may be attributed to the systemic preload, which carries cancer cells. Furthermore, Saunders et al.'s hypothesis of stereotyped muscular contractions preventing metastatic seeding may contribute to the relatively reduced rates of left-sided cardiac metastasis. 

Presently, no correlation linking cardiovascular risk factors to the development of cardiac metastasis has been found [24]. Despite this, MCTs often present with symptoms of acute coronary syndrome, as they may inhibit myocardial contractility to result in arrhythmias and heart failure [14,17]. The impact of early metastatic disease on cardiac function is usually compensated for by adaptive changes, resulting in a preserved ejection fraction in heart failure [24]. Nevertheless, MCT patients are rarely treated for heart failure [24], possibly contributing to the poor prognosis of the metastatic disease. Overall, if a previous primary carcinoma is known, the sudden appearance of such symptoms should raise a strong suspicion of metastatic progression in patients [14]. 

*Prognosis and Management of Different Cardiac Tumours* 

Cardiac tumours, including PCTs and MCTs, are often diagnosed following a high clinical suspicion of presenting symptoms and characteristic imaging findings. The transthoracic electrocardiogram (TTE) is commonly utilised to visualise tumour location, size and morphology. Other imaging modalities include CT, MRI [13,14,15] and transoesophageal echocardiography [23]. However, histopathological diagnosis is the agreed gold standard to confirm tumour subtype, often achieved after tumour resection [14,16,18,20,21,22,23,24,25]. MRI findings are increasingly informative for pre-surgical planning and assessing the identity of the tumour before histological confirmation [21].

Primary Cardiac Tumours

Cardiac tumours possess the potential to present asymptomatically (up to 12%) or cause distressing symptoms based on their size, location, mobility, degree of invasiveness and embolisation within the heart [14,15,19]. Ekmektzoglou et al. differentiate the symptomatic presentation of the neoplasms based on their dominance over the cardiac or whole body system. Systemic symptoms include fever and myalgia, and are prevalent in a third of patients. The release of inflammatory mediators and markers, such as interleukin 6, by neoplastic cells is responsible for the nonspecific systemic symptoms and may pose a diagnostic delay [14,15,19]. On the other hand, predominant cardiac symptoms mimic primary cardiac diseases such as congestive heart failure and arrhythmias [14,15].

Those with PCT usually present with a range of cardiac predominant symptoms compared to those with metastatic disease. Reportedly, the most clinically apparent symptoms are those of heart failure (HF), such as paroxysmal nocturnal dyspnoea and orthopnoea. Secondary to the cardiac symptoms are symptoms of cerebral and coronary vascular emboli [15,17,18]. However, Barreriro et al. state that the most frequent presentations for PCT are symptoms of cardioembolic stroke (25.8%) and congestive HF (25.8%) equally [21]. Nonetheless, studies share a similar consensus on the prevailing presentations of PCTs. 

The most common presentation of the malignant PCT UHGPS is dyspnoea, symptomatic in 74% of cases, followed by palpitations. Similar to PBCTs, embolic symptoms may also affect several organ systems. Additionally, angiosarcomas are another subtype of PMCTs and possess characteristic aggressive features [17]. By the time angiosarcomas are identified, metastasis to the brain and liver can be expected, with literature expressing rates of up to 47% to 89% in symptomatic patients [19]. The combination of a right atrial mass with an accompanying pericardial effusion is an unsettling indication for a probable angiosarcoma diagnosis [17]. 

General Treatment of Cardiac Tumours 

High mortality rates in cardiac tumours have been attributed to symptoms of progressive cardiac failure caused by the lesions [23]. PCTs, benign or malignant (without metastasis), are generally treated by surgical excision to improve the overall prognosis [14]. The extensiveness of surgical evisceration directly contributes to this prognosis [22]. Ekmektzoglou et al. have noticed a recurrence rate as low as 6% in PCTs after excision [15], with the recurrence of PBCTs such as myxomas being extremely scarce [14]. However, recurrence may occur more frequently in cases where poor resection techniques have been used, familial syndromes have been recognised, or the primary originating chamber has been falsely identified [14].

Differing tumour histology may further contribute to the increased likelihood of recurrence in resected cancerous lesions, as most patients in Chen et al.'s study on sarcomas experienced [25]. Multi-modality treatment consisting of surgery and chemotherapy has been shown to increase the overall survival time as compared to single-modality treatment [25]. Alternative curative treatment options include cardiac transplantation, which is often advised in the setting of obstructive masses, resulting in symptoms of arrhythmias and poor blood flow [15]. Positively, the overall trend in the past five years reflects an increase in survival rates within cardiac oncology, possibly due to the improvement in medical and surgical treatment [20]. 

A conservative approach may be offered to a limited cohort of patients. These patients require a diagnosis of small PBCTs (such as fibroelastomas) without septal defects within the atria, or a diagnosis of advanced metastatic disease [14]. However, those opting for a conservative approach face the risk of significantly reduced survival time, with an average of 2 months, typically due to the catastrophic embolisation of cardiac masses. Hence, surgery is the recommended treatment to optimise prognosis in PCTs, with debulking techniques tailored for rapidly progressive tumours, allowing palliative care [14,21]. 

Treatment of PMCTs 

Cardiac sarcomas, the most common PMCT, are often challenging to treat surgically due to the habitual late presentation of the disease. Agaimy et al. noted an average survival time of 24 months after excising the sarcoma [23]. Surgical excision with negative margins added 11 months to the survival time produced by excision with positive margins [20]. These tumours have a better prognosis if they originate from the left atrium. However, the infiltrative nature of these tumours challenges surgical guidelines by limiting complete excision with wide margins. In such cases, Butanty et al. found that adjunctive low-dose radiotherapy pre- or postoperatively decreased the extent of tissue invasion [14]. However, radiotherapy may not positively contribute to the overall prognosis if metastasis has already occurred [26]. Prior studies indicate no clear benefit of using adjunctive chemotherapy for sarcomas. Nonetheless, adjunctive therapy should still be considered due to the high recurrence rates noted in the literature and may contribute positively to the prognosis if metastasis has occurred [14, 18, 26]. 

The Prognosis of Primary Cardiac Tumours and Their Contributing Factors

Primary cardiac tumours often have a better prognosis and a lower recurrence rate than metastatic ones [15,25]. Metastatic cardiac tumours are frequently challenging to treat and fully resect due to their infiltrative nature [14,23]. Chen et al.’s study on patients with primary sarcomas (PMCT) identified factors such as age (less than 65), localised disease and surgical resection to impact the prognosis positively [25]. Surgical debulking of isolated lesions in the heart further contributes as a possible prognostic advantage [17]. Moreover, tumour size is often considered a prognostic factor in most cardiac lesions, unless patients are receiving chemotherapy. It is imperative to mention the lack of constructed guidelines for tumour sizing and staging, which prevents the formation of direct correlations between tumour size cut-offs and prognostic outcomes [26].

On the other hand, high pKi67 expression and haemorrhage were linked to an overall worse prognosis [12,13]. Liu et al. found that haemorrhaging significantly affected the extent of cellular heterogeneity in myxomic tumour cells [13]. Angiosarcomas are a good example to support this correlation further, as they are also haemorrhagic and aggressive, with increasing mitotic and necrotic activity, resulting in an overall poor prognosis. These similarities between myxomas and angiosarcomas often lead to misdiagnosis between the two tumours [19], which can drastically impact a patient's prognosis as myxomas are benign and angiosarcomas are malignant in nature. 

It's important to note that About et al. did not find the development of metastasis from a PCT to be statistically significant in affecting patient survival rates; likewise, the location of the tumour was not considered a significant prognostic factor [12,17]. Estimating the overall prognosis for patients with tumours, such as primary sarcomas (PMCT), can be accurately achieved if independent prognostic factors are combined with tumour grading [23,25]. 

Metastatic Cardiac Tumours 

MCTs have been identified to possess a more violent biological nature, resulting in the extensive technical skills needed to remove such masses surgically [15,17]. Resection of extensive infiltrating metastatic disease is typically unachievable and requires wonted secondary surgical procedures to repair the resected anatomy [14,22]. However, treating metastatic disease has been linked with an increased mean survival time of 27.3 months as compared to 6.6 months without treatment, as seen in metastatic sarcomas. Lin et al. concluded that metastatic disease responsive to treatment, such as chemotherapy, had an extended therapeutic effect on the primary lesion. Therefore, highlighting the benefits of treating MCTs [24]. However, individuals with MCT routinely require palliative care, as up to 78% of patients face reduced blood flow through mechanical interference [15,17]. Palliative care aims to improve the patient's quality of life through symptom management and to delay the frequency of symptoms [17]. The prognosis for MCTs remains challengingly low with an expected survival rate of less than 25%, one year after the diagnosis [15]. 

Therefore, Ekmektzoglou et al. warn physicians of patients with high-risk tumours, such as lung tumours or melanomas, and expect them to be aware of any diversification in the baseline cardiac rhythm, as it could present as an early sign of potential cardiac metastasis. Additionally, pericardial malignancy should be considered as a differential diagnosis in those with a pre-existing cancer diagnosis and symptoms of refractory heart failure or recurrent/major effusions [15,17,19]. 

Neonates 

In neonates, fatal outcomes from cardiac tumours have been identified, including hydrops fetalis resulting in stillbirth or sudden cardiac death postnatally, possibly without prior clinical warning signs. Nonetheless, a spectrum of outcomes exists based on the histopathological nature and location of the cardiac tumour. Isaac et al. highlight the poor prognosis associated with cardiac tumours and comorbid heart malformations in neonates. Additionally, the rare diagnosis of MCT in neonates is associated with a bleak survival rate, often with no survivors [16]. 

However, rhabdomyomas are known to be the most prevalent PCT within neonates and are known to regress spontaneously. If symptoms of rhabdomyomas, such as ventricular tachycardia, are controlled during infancy, an excellent prognosis can be achieved in early childhood. Note that hemangiomas behave in a similar regressive manner. Additionally, some neonatal cardiac tumours, such as teratomas, do not possess self-limiting properties but still sustain a good prognostic outcome once excised surgically [16,17]. Therefore, while cardiac tumours can be catastrophic in neonates, they are more likely to have a positive outcome.

Limitations

Due to the low prevalence of cardiac tumours, some of the studies included within this systematic review may have offered skewed research outcomes in terms of gender prevalence, tumour location and incidence rates. Additionally, a limited number of published papers exist to explain the cardiac microenvironment, where others have extended their results of skeletal muscle action to hypothesise the role of the cardiac TME in cancer development. Therefore, it is recommended that more studies be conducted to explore the behaviour of cardiac muscle cells on a microscopic level to further explore immunological cancer markers and differences in neoplasms. Finally, studies using a larger cohort of patients are recommended to confirm epidemiological and biological patterns of different cardiac tumours.

## Conclusions

Overall, this review identifies and analyses varying factors contributing to the differences in the cardiac tumour microenvironment and those involved in the prognostic outcome. Correlating with previous scholarly articles, metastatic cardiac tumours are more prevalent than primary cardiac tumours. This occurs due to the tight regulations in cellular division in cardiac myocytes as compared to other organ systems, increasing the resistance to PCT development. Theories highlighting high blood flow to cardiac muscle and its stereotyped contractile motion may explain the preferential location of metastatic cardiac tumours. Additionally, the role of mutations in tumour-suppressing genes was found to increase the risk of neoplasm formation by bypassing the highly controlled cellular environment. Prognostic factors were divided into tumour properties and medical intervention; these included the size, location, type of tumour, and the type and extent of intervention. This article examines cardiac neoplasms from their cellular origin, reflecting on genomic mutations to explain probable causes for the low communal prevalence. Gaining a better understanding of this topic may enable cardiac oncologists to target the root cause of tumour development.
